# The effect of deliberative process on the self-sacrificial decisions of utilitarian healthcare students

**DOI:** 10.1186/s12910-022-00769-w

**Published:** 2022-03-19

**Authors:** Yongmin Shin, Seungmin Kim, Do-Hwan Kim, Seunghee Lee, Minhae Cho, Jungjoon Ihm

**Affiliations:** 1 Dental Research Institute, School of Dentistry, Seoul National University, Seoul, Korea; 2 Department of Medical Education, College of Medicine, Hanyang University, Seoul, Korea; 3 Department of Medical Education, College of Medicine, Seoul National University, Seoul, Korea; 4 Interdisciplinary Program in Cognitive Science, Seoul National University, Seoul, Korea; 5 School of Social Work, University of Memphis, Memphis, USA

**Keywords:** Medical ethics, Utilitarian judgments, Dual process model, Cognitive reappraisal, Deliberative process

## Abstract

**Background:**

The COVID-19 pandemic has highlighted prosocial behavior as a professional healthcare core competency. Although medical students are expected to work in the best interests of their patients, in the pandemic context, there is a greater need for ethical attention to be paid to the way medical students deal with moral dilemmas that may conflict with their obligations.

**Methods:**

This study was conducted in the spring semester of 2019 on 271 students majoring in health professions: medicine, dentistry, and veterinary medicine. All participants provided informed consent and completed measures that assessed utilitarian moral views, cognitive reflections, cognitive reappraisal, and moral judgment.

**Results:**

The healthcare-affiliated students who scored higher on the instrumental harm subscale in the measurement of utilitarian moral views were more likely to endorse not only other-sacrificial actions but also self-sacrificial ones for the greater good in moral dilemma scenarios. In particular, those engaged in deliberative processes tended to make more self-sacrificial judgments. The mediation analysis also revealed that the effect of deliberative processes on self-sacrificial judgments was mediated by cognitive reappraisal.

**Conclusions:**

These findings suggested that cognitive reappraisal through deliberative processes is involved when the students with utilitarian inclination make prosocial decisions, that it is necessary to consider both moral views and emotional regulation when admitting candidates, and that moral education programs are needed in the healthcare field.

**Supplementary Information:**

The online version contains supplementary material available at 10.1186/s12910-022-00769-w.

## Background

The COVID-19 pandemic has highlighted prosocial behavior as a professional healthcare core competency [1]. Although prospective medical doctors are supposed to be trained to work in their patients’ best interests, in the pandemic context, greater ethical attention needs to be paid to the methods used by medical students in dealing with moral dilemmas that may conflict with their obligations. Morality has been significantly challenged by the COVID-19 pandemic, especially when there is a high risk of exposure to the COVID-19 infection; therefore, students are being confronted with the moral dilemma of either self-sacrificing themselves to treat patients or refusing duty to protect their personal safety.

Many moral judgment studies have investigated sacrificial moral dilemmas in hypothetical situations in which decisions need to be made to endorse the sacrifice of innocent individuals in order to maximize the welfare of many other people [2, 3]. A dual moral judgment process model based on two distinctive cognitive systems, System 1 and System 2, was proposed to explain the cognitive processes involved in hypothetical moral judgments [4]. Dual-process theory states that decision-making is influenced by the interplay of the two cognitive systems [5]. System 1 is an automatic, intuitive, and affective process, and System 2 is a slow, controlled, and deliberative process. Applying this framework to the context of moral judgment, in System 1, when an individual is considering whether to endorse harmful actions to maximize welfare, negative emotional reactions to the causing of harm result in a rejection of the actions, whereas in System 2, overriding these negative emotional reactions motivates an endorsement of the actions. Therefore, System 1 involves deontological judgment, whereas System 2 involves utilitarian judgment.

Several previous studies on utilitarian judgments have reported a positive relationship between utilitarian responses to moral dilemmas and negative personality traits, such as psychopathy or egoism, and have considered the utilitarian responses to reflect negative personality traits [6–9]. This is because genuine utilitarian moral concerns have not been distinguished from the negative aspects of utilitarian moral thinking which involves the use of a person as a means to an end in classical sacrificial moral dilemmas. Some studies have recently demonstrated the appositive aspects of utilitarian moral views that contribute to impartial maximization of everyone’s well-being [10, 11]. In addition, other studies have suggested that sacrificial utilitarian judgments reflect moral concerns and are not associated with antisocial tendencies or egoism [12, 13]. Although recent studies have explored the associations between utilitarian motivation and judgments and prosocial aspects [11–13], to the best of our knowledge, there have been no studies that have investigated the moral decisions of utilitarian-inclined healthcare-affiliated students. Therefore, to elucidate the moral and prosocial aspects of utilitarian decisions in professional healthcare education, it is important to identify the relationships between utilitarian student decisions and prosociality.

As System 2 decisions affect utilitarian judgments in the dual-process model, it could be surmised that cognitive abilities play a significant role in moral judgments. In general, because System 2 is more analytical and rational, its application possibly requires greater cognitive effort. However, the relationship between the cognitive underpinnings of healthcare-affiliated students with utilitarian inclinations and their prosocial decisions remains unexplored in medical ethics studies. Therefore, by examining these utilitarian cognitive underpinnings using moral dilemmas, a path to moral student decisions can be identified. In particular, as moral judgments are influenced by an emotional response to given situations or scenarios [14, 15], emotional regulation may have a significant cognitive effect on utilitarian judgments.

Emotional regulation involves shaping when and how emotions are experienced and expressed [16, 17]. Therefore, emotional regulation comprises a cognitive reappraisal (CR) and expressive suppression (ES). CR is when individuals ponder a situation in a way that changes their emotional responses, whereas ES is when individuals reduce their emotional behavior when emotionally aroused. As intuition is a cognitive process that has strong relationships with emotional responses [4], overriding intuitive responses, as in System 2, may be similar to CR. Various studies have found that a higher CR was significantly related to less deontological and immoral judgments and more deliberative judgments [18–20]. Therefore, to better understand the underpinnings of utilitarian students when making moral judgments, an examination of both cognitive ability and CR is necessary.

This study examined the prosocial and cognitive underpinnings of utilitarian students majoring in health professions, who were defined as people who tended to make sacrificial utilitarian judgments in our moral dilemma scenarios, not as people who had philosophical utilitarian principles. Utilitarian judgment appears to be based on cost–benefit reasoning and the minimization of total harm for the greater good [12]. Moreover, utilitarian judgments are predicted by aversion to bad outcomes, derived from genuine moral concern for others’ well-being [13]. In this regard, it is expected that people with utilitarian inclinations will be likely to sacrifice themselves to prevent harm to others. However, in responses to moral dilemmas, it is difficult to grasp the motivation for judgments and moral views. With reference to our research goal, the response to moral dilemmas, including self-sacrifice, and utilitarian motivations, instrumental harm and impartial beneficence, were measured together, to identify the relationships between utilitarian responses and utilitarian motivations.

Moreover, as found in previous dual-process model studies [21–23], it was anticipated that participants engaging System 2 and scoring high on the CR subscales would be likely to accept harmful actions for the greater good would be likely to accept harmful actions for the greater good and, based on two variables that share a common cognitive control, namely, intuition and emotion, that CR mediated the relationship between System 2 and utilitarian decisions. Previous studies have demonstrated that decisions made based on personal preferences are more likely to be System 1 decisions, whereas those made that are contrary to personal preferences are more likely to be System 2 decisions [24–26]. Therefore, it was hypothesized that study participants who engage System 2 and score high on the CR subscales are likely to make self-sacrificial decisions for the greater good.

## Methods

### Participants

To detect a medium-sized effect (*f* = 0.25) with 95% power, a priori power analysis was conducted using the G*Power software to calculate the minimum sample size, which was found to be 188. For this cross-sectional study, a sample of 271 undergraduate students, aged 19–35 (*M* = 22.94, SD = 2.63), majoring in health professions at the Seoul National University were recruited in the spring semester of 2019 (121 (44.7%) from medicine, 106 (39.1%) from dental medicine, and 44 (16.2%) from veterinary medicine; 127 (46.8%) of them were female). All participants voluntarily gave written informed consent before participation.

### Measures and materials

*Emotion regulation questionnaire* (ERQ) [27]. The ERQ is a 10-item self-report questionnaire comprising two subscales that assess two different emotional regulation strategies, reappraisal, which has items such as “When I want to feel less negative emotion such as sadness or anger, I change what I’m thinking about,” and suppression, which has items such as “I control my emotions by not expressing them,” the subscales for which comprise six and four items, respectively. The 10 items are rated on a 7-point Likert scale ranging from 1 (strongly disagree) to 7 (strongly agree). In the present study, both subscales were found to have good reliability (CR: *α* = 0.87, ES: *α* = 0.73).

*Cognitive reflection test* (the original CRT version [28] and CRT 2 [29]). The original version of the three-item CRT, which has items such as “If it takes 5 machines 5 min to make 5 widgets, how long would it take 100 machines to make 100 widgets?,” measures cognitive traits to determine whether respondents depend on an intuitive process, such as System 1, or a deliberative process, such as System 2. This study added four recently developed items to CRT 2 (e.g., “If you’re running a race and you pass the person in second place, what place are you in?”), and a composite score for the original CRT version and the CRT 2 was calculated, with the total possible score being 7. As the CRT questions induce responders to answer intuitively, correct answers are only possible with cognitive reflection; therefore, students who rely on intuitive processes tend to perform poorly in the CRT.

*Oxford utilitarianism scale* (OUS) [11]. The OUS comprises nine items across two subscales: impartial beneficence (IB: 5 items) and instrumental harm (IH: 4 items). The OUS measures the overall pattern of utilitarian moral views, with the IB examining the degree of endorsement given to an impartial maximization of the greater good even at the cost of personal self-sacrifice (e.g., “From a moral point of view, we should feel obliged to give one of our kidneys to a person with kidney failure since we don’t need two kidneys to survive, but really only one to be healthy.”) and the IH reflecting the willingness to cause harm to bring about the greater good (e.g., “It is morally right to harm an innocent person if harming them is a necessary means to helping several other innocent people.”). The nine items are rated on a 7-point-Likert scale ranging from 1 (strongly disagree) to 7 (strongly agree). In the present study, both subscales had acceptable reliability (IB: *α* = 0.62, IH: *α* = 0.76).

*Modified moral dilemmas* (Additional file 1: Supplementary information file). Three moral dilemma scenarios were selected from a previous study [30]. The original dilemmas were then modified because if several dilemmas were employed for each experimental condition, the unique dilemma content was thought to possibly confound the respondents’ judgments. Including self-interest seeking also meant that only minimal changes needed to be made to the original scripts. Therefore, the dilemmas were carefully selected to ensure that the healthcare-affiliated respondents’ prior knowledge would not interfere with the moral conflicts in the dilemmas. The selected dilemmas were a crying baby, a burning building, and a submarine incident. The common features in these three dilemmas were as follows:

1. There is an event that threatens the survival of a group of people.
2. In this group of people, there is an isolated individual, that is, a minority.
3. Any intervention involves a lethal sacrifice of the minority for the remaining majority.
4. The minority always survives unless the intervention takes place.
5. In the absence of the intervention, the majority always fails to survive the given event.

The self-interest element was embedded in the dilemmas in two conditions; therefore, for each dilemma, there were three conditions, which included the self-interest neutral (control) condition:

1. Neutral condition: In this condition, participants take an observer’s point of view, with the pronouns suggesting any personal involvement in the scenario, such as “you are holding a baby,” being replaced with neutral terms, such as “a parent is holding a baby.”
2. Self-as-minority condition: In this condition, participants play the role of the minority in the particular scenario.
3. Self-as-majority condition: In this condition, participants play the role of one of the majorities.

Contrary to the neutral condition, a self-interest element that added another moral conflict layer to the existing utilitarianism versus deontology conflict was imposed on the respondents in the two experimental conditions. For each dilemma, the respondents were first asked whether they agreed or disagreed with the intervention. They again answered a question on the extent to which they agreed with their choices on a scale of 1–3. The scores for these questions were converted to a 6-point Likert scale, ranging from 1 (strongly against the intervention) to 6 (strongly supporting the intervention). A composite index for each condition, that is, neutral, self as minority, and self as majority, was an aggregation of the three scores from each dilemma condition, with the total possible score for each condition being 18. In the present sample, the dilemmas were found to have good reliability (*α* = 0.80).

### Procedures

The healthcare-affiliated students responded using the online survey tool LimeSurvey (Hamburg, Germany: LimeSurvey Project). In the first part of the survey, they completed the OUS, ERQ, and CRT, after which they completed the modified sacrificial dilemmas. The moral dilemmas were presented in batches, with the neutral scenarios being proffered before the modified versions were introduced. A counterbalancing methodology was employed to randomly assign the respondents to two different experimental conditions: one group completed variations A and B after the neutral scenario (N–A–B), and the other group completed variations B and A after the neutral scenario (N–B–A). Finally, demographic information was collected. The PROCESS macro for mediation analysis [31] in SPSS 25.0 (SPSS Inc., Chicago, IL, USA) was employed for the analysis.

## Results

### Descriptive statistics and correlation analyses of the variables

The descriptive statistics revealed that in all conditions, the mean for the endorsement of the minority-sacrifice was above the median total score in each condition (Table 1). The correlational analyses revealed that there were positive correlations between all sacrificial dilemmas. The self-as-minority condition was significantly related to all major variables at *p* < 0.01, whereas the neutral and self-as-majority conditions were only associated with IH at *p* < 0.01. Correlations were also found between CRT and CR at *p* < 0.01.

**Table 1:** Means, standard deviations, and correlations for the major variables

Variables	*M* (SD)	1	2	3	4	5	6
1. Cognitive reflection test	5.41 (1.38)						
2. Cognitive reappraisal	28.67 (5.91)	.18**					
3. Impartial beneficence	17.24 (4.39)	− .04	.01				
4. Instrumental harm	12.45 (3.91)	.01	.12*	.25**			
5. Dilemmas (neutral)	10.56 (2.23)	.06	.01	− .03	.39**		
6. Dilemmas (self-as-minority)	10.66 (2.75)	.19**	.20**	.17**	.22**	.44**	
7. Dilemmas (self-as-majority)	10.96 (2.81)	.01	− .03	− .09	.36**	.75**	.32**

*p < .05; **p < .01

### Utilitarian judgments

Before the main analyses, a normality test and homogeneity of variance test were performed. Levene’s statistic was not significant (Neutral condition: *F*(1, 269) = 0.087, *p* = 0.769; Self-as-minority condition: *F*(1, 269) = 0.137, *p* = 0.712; Self-as-majority condition: *F*(1, 269) = 0.330, *p* = 0.566), which indicates that the assumption of the homogeneity of variance was not violated, while the Kolmogorov‒Smirnov statistic was significant for all the conditions (all the conditions: *p* < 0.001), which indicates that the normality assumption was violated. However, ANOVA and linear models were found to be robust to the violation of normality [32, 33], and thus main analysis proceeded.

**Fig. 1:**
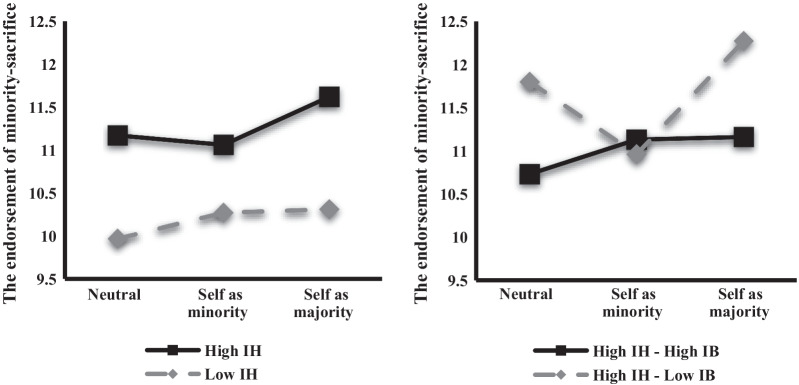
Endorsement of minority-sacrifice in the three dilemma conditions in the IH and IB groups. IH, instrumental harm subscale; IB, impartial beneficence subscale

To examine the IH, IB, and dilemma scenario condition effects on the utilitarian judgments, a 2 × 2 × 3 mixed-design ANOVA was conducted, for which the IH and IB groups were divided using mean splits. The results indicated a significant main effect for the IH group (*F*(1, 265) = 19.97, *p* < 0.001, $\eta_{p}^{2}$ = 0.070), an interaction effect for the IH and IB groups (*F*(1, 265) = 6.88, *p* = 0.009, $\eta_{p}^{2}$ = 0.025), and an interaction effect for the IB group and the dilemma conditions (*F*(1.50, 397.11) = 4.82, *p* = 0.016, $\eta_{p}^{2}$ = 0.018) (Table 2 and Fig. 1). The simple effects analysis using Bonferroni pairwise comparisons revealed that the high-IH group (*N* = 134) was significantly more likely to endorse the minority-sacrifice over all conditions than the low-IH group (*N* = 137), *p* < 0.05. While no difference was observed in the endorsement between the two IB groups (high: *N* = 132, low: *N* = 139), the low-IB group was significantly more likely to accept the minority-sacrifice in the self-as-majority condition than in the other conditions.

**Table 2:** Mixed-design ANOVA for the moral judgment differences between the IH and IB groups

		SS		*df*		MS		*F*		η^2^_*p*_
*Between group*										
Instrumental harm (*A*)		243.36		1		243.36		19.97***		0.070
Impartial beneficence (*B*)		0.44		1		0.44		0.36		0.000
*A* × *B*		83.87		1		83.87		6.88**		0.025
Error		3228.74		265		12.18				
*Within group*										
Dilemma condition (*C*)		2.94		1.50		1.96		0.42		0.002
*A* × *C*		16.15		1.50		10.77		2.32		0.009
*B* × *C*		33.58		1.50		22.41		4.82*		0.018
*A* × *B* × *C*		7.12		1.50		4.75		1.02		0.004
Error		1847.95		397.11		4.65

The analysis of the total scores for each dilemma condition revealed that the group high on IH but low on IB (HH–LB, *N* = 55) had a significantly higher inclination to endorsing sacrificial harm than the group high on both IH and IB (HH–HB, *N* = 79) and the group low on both IH and IB (LH–LB, *N* = 84), *ps* < 0.05 (Table 3). However, no significant difference was observed in the endorsement between the two groups high on IB (LH–HB, *N* = 53) or between the two groups low on IH. Even though the interaction effects of IH, IB, and the conditions were not significant, the pairwise comparisons revealed that the HH–LB group tended to more significantly endorse the minority-sacrifice than the HH–HB group only in the neutral and self-as-majority conditions, *p* = 0.004. However, there was no significant endorsement difference between the two groups that were low on IH.

**Table 3:** Post hoc comparisons for the moral judgment difference between the IH and IB groups

Comparison		Mean difference	SE
IH	IB		
High IH*High IB	High IH*Low IB	− .705*	.356
High IH*High IB	Low IH*High IB	.470	.360
Low IH*Low IB	Low IH*High IB	− .609	.354
Low IH*Low IB	High IH*Low IB	− 1.784*	.351

Age and gender were adjusted

IH, instrumental harm subscale; IB, impartial beneficence subscale; SE, standard error

*p < .05

### Effects of deliberation and CR on self-sacrificial judgments for the greater good

**Fig. 2:**
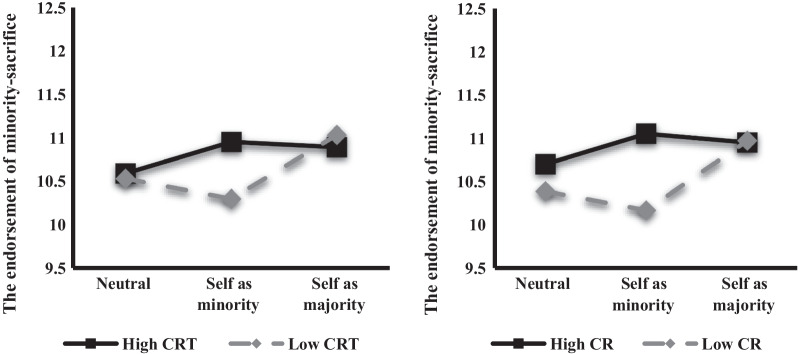
Endorsement of minority-sacrifice in the three dilemma conditions in the CRT and CR groups. CRT, cognitive reflection test; CR, cognitive reappraisal

The same mixed-design ANOVA was conducted (within-subjects, the three dilemma conditions; between-subjects, CRT or CR using mean split). Although the main effects were not significant, the interactions were significant: CRT (*F*(1.49, 398.80) = 3.44, *p* = 0.047, $\eta_{p}^{2}$ = 0.013), CR (*F*(1.49, 398.83) = 3.84, *p* = 0.034, $\eta_{p}^{2}$ = 0.014) (Fig. 2). The simple effect analysis revealed that the high-CRT (*N* = 151) and high-CR (*N* = 150) groups were significantly more likely to endorse a minority-sacrifice than the low-CRT (*N* = 120) and low-CR (*N* = 121) groups only in the self-as-minority condition (CRT: *p* = 0.045, CR: *p* = 0.009).

**Fig. 3:**
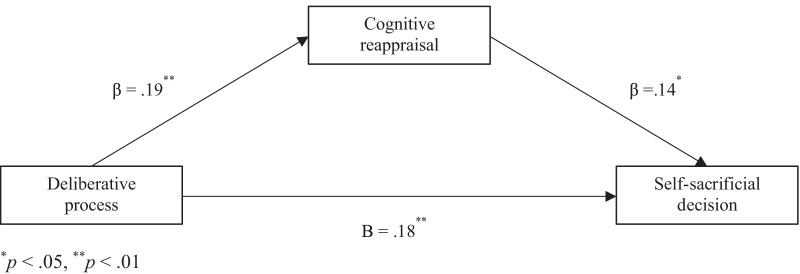
Mediating effects of cognitive reappraisal on the relationship between deliberative processes and self-sacrificial decisions

A mediation analysis controlling for age, gender, IH, and IB was conducted to identify whether CR mediated the relationship between cognitive reflection and utilitarian decisions, for which the dependent variable was the decision in the self-as-minority condition as both CRT and CR only had effects on the decision. It was found that CRT made positive significant predictions about self-sacrificial decisions (*b* = 0.42, *p* < 0.001) (Fig. 3). The predicted indirect effect through CR was also found to be statistically significant (*a* × *b* = 0.054, 95% CI [0.031, 0.129]).

## Discussion

This study employed modified moral dilemmas to examine the prosocial attributes of utilitarian healthcare-affiliated students. It was found that the students with utilitarian inclinations were found to endorse both other-sacrifice and self-sacrifice for the greater good and that CR mediated the relationship between cognitive reflection and self-sacrifice judgments. These results supported the hypothesis that utilitarian student moral reasoning tends to be prosocial and is affected by System 2 and CR.

Specifically, in the three dilemma conditions, the high-IH group endorsed significantly more utilitarian judgments overall than the low-IH group. The total scores for each condition indicated that the high-IH–low-IB (HH–LB) group endorsed significantly more utilitarian judgments than the other groups. This finding could have been due to the IB subscale characteristics, which reflect self-sacrifice for the greater good and are positively correlated with empathic concern and altruistic judgments [11]. In other words, those low on IB are more likely to endorse other-sacrifice and less likely to endorse self-sacrifice. The findings supported this notion as they indicated that the low-IB group tended to more significantly endorse sacrificial harm in the self-as-majority condition than in the other conditions. Therefore, it could be speculated that the HH group’s low IB intensified their utilitarian judgments.

However, no significant differences were observed between the HH–LB and HH–HB groups in the endorsement of the utilitarian decisions in the self-as-minority condition, indicating that the HH–LB group made as many self-sacrificial judgments as the HH–HB group and that the high-IH group’s self-sacrificial judgments may have been more affected by other factors than empathy or altruism. The modified dilemmas used in this study were related to sacrificial harm, whereas the materials used to measure prosocial aspects in previous studies [8, 11, 12] were related to helping others. As no relationship between sacrificial utilitarian judgments and prosocial aspects was examined in these studies, this study’s findings suggest that the motivation for self-sacrificial judgments may have originated from moral concerns about harm minimization.

Contrary to the high-IH group, the low-IH group was consistently found to be less likely to accept the minority-sacrifice in all three conditions. A confusing result was that the low-IH group was less likely to endorse the minority-sacrifice in the self-as-minority condition and was more likely to accept the other-sacrifice for self-interest, whereas the low-IH group was less likely to accept the other-sacrifice for the greater good in the other conditions than the high-IH group. It is possible that this result was due to omission bias, that is, the harm caused by the action was perceived to be worse or less moral than the harm caused by inaction [34, 35]. As decisions based on a sensitivity to moral norms and decisions based on a general preference for inaction are qualitatively different [36], it was assumed that the low-IH group’s decisions may have been biased to avoid the negative emotions resulting from a sacrificial judgment, which implied that the low-IH group may not have been more inclined toward following the moral norms prescribing that individuals should not harm others compared with the high-IH group.

As expected, in the cognitive underpinnings of the utilitarian decisions, the decision patterns for the CRT and CR groups were the same in all dilemma conditions. This result supported the assumption that the two variables share a common mechanism, that is, the deliberative process. However, unexpectedly, CRT and CR were only found to have an effect on decisions in the self-as-minority condition, not in the neutral and self-as-majority conditions, which differed from the dual-process theory for moral judgments. Inconsistent findings with this theory have also been previously reported [37–39].

A hybrid dual-process model is proposed as an alternative to explain these inconsistent results [40, 41]. In this model, both deontological and utilitarian intuitions are simultaneously generated through System 1. If one of the two intuitions is evidently stronger, then intuition is chosen as the initial response. When the relative difference in the strength between the two intuitions is small, System 2 would be used to override one of the conflicting intuitions and change the initial response. When these principles were applied to our findings, it was concluded that most students made intuitive utilitarian responses to the dilemmas in the neutral and self-as-majority conditions regardless of their engagement in System 2 or CR because their utilitarian intuition was stronger than their deontological intuition.

However, depending on the engagement of these two variables, the decisions differed in the self-as-minority condition, indicating that most experienced internal conflicts at the System 1 level as the difference in the relative strength between the prosocial and self-interest intuitions was small. This could be seen as a conflict between preference and non-preference, that is, people prefer self-beneficial situations and intuitively or emotionally respond when making decisions based on personal preferences [24–26]. In the current study, the more the participants engaged in System 2 or CR, the more they tended to make self-sacrificial decisions in the self-as-minority condition, that is, those high on CRT and CR controlled their preference for the greater good. This was supported by the mediation analysis finding that CR partially mediated the relationship between CRT and self-sacrificial decisions, which may suggest that regardless of the IH and IB scores, this is the general cognitive characteristic of prosocial utilitarian students. Even though it was found that System 2 has effects on utilitarian judgments, such effects are limited to the prosocial aspects of utilitarian judgments. Therefore, students’ ethical awareness and moral reasoning should be monitored in medical curricula to develop their deliberative processes and lessen the cognitive errors that could impact patient care and safety.

Understanding students’ responses to moral dilemmas can assist in designing pedagogical activities that motivate reflection and reasoning on ethical healthcare issues. These activities should be coordinated under the guidance of a non-judgmental facilitator who is prepared to listen to the students’ thoughts and feelings and provide insights for their professional development [42]. One educational intervention could be to include bioethics courses in medical curricula; however, a more effective course of action could be to facilitate educational interventions that encourage medical instructors to share their values and moral reasoning as well as their medical knowledge and skills by engaging in problem solving of real cases that pose moral and ethical challenges. In particular, work with critical incidents has been found to be an effective way of dealing with the real-life ethical dilemmas of medical students [43].

This study shows that both cognitive ability and emotional factors play an important role in making self-sacrificial decisions in moral dilemmas. It has been pointed out that the current medical school curriculum tacitly supports the attitude of distancing oneself from one’s emotions and overlooks the importance of education on emotions [45, 46]. A formal educational program that emphasizes the role of emotions in moral judgment and the importance of ability to control one's emotion needs to be supplemented. The primary aim of these courses should include cultivating the ability to recognize one’s emotion and expressing them in appropriate language. Specifically, it is necessary to consider a teaching method that encourages expressing one’s emotion “out loud” through spoken words (e.g., group discussion) or writing (e.g., reflective writing) [47]. These activities should be coordinated under the guidance of a non-judgmental facilitator who is prepared to listen to the students’ thoughts and feelings and provide insights for their professional development [37]. Throughout the course, students will benefit from opportunities to realize that not only they but also their colleagues and patients may face emotionally charged clinical situations. This could help improve their ability to analyze the underlying causes of a given situation more systematically, especially when others’ moral judgments do not match their own.

Problem-based learning, which typically uses specific case scenarios to teach basic and clinical medicine through self-directed and cooperative learning, has been proposed as an effective way of providing ethics education for healthcare students [49]. Similarly, it has been found that working with critical incidents could be an effective way of dealing with the real-life ethical dilemmas [38]. This kind of education will give students an opportunity to practice ethical decision-making in a safe environment without risk to the patient and to discuss the different perspectives regarding moral reasoning. In this process, students will recognize the diversity of each other’s utilitarian tendencies and emotion regulation strategies, which ultimately help them to understand that these differences are linked to prosocial decision-making. Considering the diverse areas of focus of participants in this study, it seems possible to organize interprofessional education programs targeting students in different healthcare disciplines simultaneously [48]. In this case, the educational effect would be more significant if it were possible to attain the participation of instructors in various health professions who can add diversities in their values and moral reasoning, as well as their medical knowledge and skills.

This study has several strengths and limitations. To the best of our knowledge, this was the first study to examine the prosocial aspects of healthcare-affiliated students with utilitarian inclinations, which was in contrast to previous studies that have mainly focused on the relationship between utilitarian judgments and negative personality traits [6–9]. This study was also the first to examine the cognitive underpinnings of the prosocial aspects of utilitarian students. The findings indicated that CR plays a significant role in making prosocial utilitarian decisions through deliberative processes. However, the number of the majority in the modified sacrificial dilemmas was not controlled or manipulated. Trémolière and Bonnefon [39] found that subjects make utilitarian decisions more intuitively when sacrificing one innocent person to save thousands of people than sacrificing one innocent person to save a few. Therefore, in the self-as-minority condition, if self-sacrifice is required to save thousands of people, then there may be a possibility that most students would intuitively accept self-sacrifice. Therefore, it is recommended that dilemma scenarios in future research be more controlled and manipulated. Another limitation of this study was that it was conducted on students from a single Asian university; therefore, the observed moral judgments need to be investigated in different sociocultural settings. Finally, this study focused on students’ moral reasoning and individual differences variables influencing it, and the classical trolley dilemma scenarios previous studies have employed were used. However, considering that the context of the scenarios may affect judgments [44], future study will need to identify whether our findings are replicated in the dilemma scenarios including medical contexts.

## Conclusion

This study not only supported the positive aspects of students’ sacrificial utilitarian judgments but also enhanced understanding on the moral judgment psychological characteristics of future healthcare professionals. Specifically, unlike previous studies that did not account for the pathway between cognitive ability and moral decisions, this study identified the cognitive underpinnings of utilitarian students’ prosocial judgments and found the role of CR to be an intermediate process. Therefore, this work has practical implications as it suggested that deliberation and emotional regulation play a significant role in moral or prosocial judgments. These findings provide evidence that it is important to consider both moral views and emotional regulation when selecting personnel and designing moral education healthcare programs.

## Supplementary Information

**Additional file 1**. List of modified moral dilemmas.

## Data Availability

The datasets used and/or analyzed during the current study are available from the corresponding author on reasonable request.

## Abbreviations

CR: Cognitive reappraisal
ES: Expressive suppression
CRT: Cognitive reflection test
OUS: Oxford Utilitarianism Scale
IB: Impartial beneficence
IH: Instrumental harm
