# Supplementary material for: The effect of deliberative process on the self-sacrificial decisions of utilitarian healthcare students
Source: BMC Med Ethics. 2022 Mar 19;23:28. doi: 10.1186/s12910-022-00769-w (PMC8933755; doi:10.1186/s12910-022-00769-w)
Supplement: Supplementary file 1 — Additional file 1. List of modified moral dilemmas. [file 12910_2022_769_MOESM1_ESM.docx]

**Additional file 1: List of Modified Moral dilemmas**

Modified Moral dilemmas (Moore, Clark, & Kane, 2008)

| *Neutral* context | |
| --- | --- |
| Crying baby | Enemy soldiers have taken over a village. They have orders to kill all remaining civilians over the age of two. Some of the townspeople have sought refuge in two rooms of the cellar of a large house. Outside the villagers hear the voices of soldiers who have come to search the house for valuables. A baby, who is with a parent amongst the villagers in the room, begins to cry loudly. The parent put his/her hand over the baby’s mouth to block the sound. If the parent removes his/her hand from the baby’s mouth the baby’s crying will summon the attention of the soldiers who will spare the baby’s life, but will kill the parent and the others hiding in both rooms. To save oneself and the others the parent must keep his/her hand on the baby’s mouth and smother the baby to death. Should the parent smother his/her baby in order to save oneself and the other townspeople?  Please choose whether you agree or disagree with the suggested action in the scenario.  Please select the extent to which you agree with your choices above. |
| Burning building | A total of seven people are trapped in a burning building. There is only one emergency exit through which everyone could escape to safety, but it is blocked by burning debris. One of the seven people in the hallway leading to the exit who has been injured is about to crawl to safety through a small hole at the bottom of the exit door. The other six people do not have time to climb through the small hole. The other six people could grab the injured person and use his/her body as a battering-ram to break through the burning blockage in the hallway that is preventing their escape. Doing this is certain to kill the injured person. However, if the rest of the people do not do this, they will die. Should the six people kill the injured person in order to save themselves?  Please choose whether you agree or disagree with the suggested action in the scenario.  Please select the extent to which you agree with your choices above. |
| Submarine | An onboard explosion has damaged a submarine, killed and injured several crewmembers. Additionally, it has collapsed the only access corridor between the upper and lower parts of the submarine. The upper section, where the most of the crewmembers are located, does not have enough oxygen remaining for all of the crewmembers to survive until they reach the surface. Only one remaining crewmember is located in the lower section, where there is enough oxygen. There is an emergency access hatch between the upper and lower sections of the submarine. If released, it will fall to the deck and allow oxygen to reach the upper section. However, a crewmember in the lower section was knocked unconscious and is lying beneath the hatch while the rest of the crew are almost out of air. If the crewmembers in the upper section shove the hatch open they will have air, but it will fall to the deck, crushing the unconscious crewmember to death. Should the crewmembers in the upper deck open the hatch and crush the crewmember below to save crewmembers in the upper deck?  Please choose whether you agree or disagree with the suggested action in the scenario.  Please select the extent to which you agree with your choices above. |
| *Self-in-minority* context | |
| Crying baby | Enemy soldiers have taken over a village. They have orders to kill all remaining civilians over the age of two. You and some of the townspeople have sought refuge in two rooms of the cellar of a large house. Outside you and the villagers hear the voices of soldiers who have come to search the house for valuables. Your baby, who is with you in the room, begins to cry loudly. You put your hand over the baby’s mouth to block the sound. If you remove your hand from the baby’s mouth the baby’s crying will summon the attention of the soldiers who will spare the baby’s life, but will kill you and the others hiding in both rooms. To save yourself and the others you must keep your hand on the baby’s mouth and smother the baby to death. Should you smother your baby in order to save yourself and the other townspeople?  Please choose whether you agree or disagree with the suggested action in the scenario.  Please select the extent to which you agree with your choices above. |
| Burning building | You and six other people are trapped in a burning building. There is only one emergency exit through which all of you could escape to safety, but it is blocked by burning debris. One of the seven people in the hallway leading to the exit who has been injured is about to crawl to safety through a small hole at the bottom of the exit door. The other six people do not have time to climb through the small hole. The other six people could grab you and use your body as a battering-ram to break through the burning blockage in the hallway that is preventing their escape. Doing this is certain to kill you. However, if the rest of the people do not do this, they will die. Should the six people kill you in order to save themselves?  Please choose whether you agree or disagree with the suggested action in the scenario.  Please select the extent to which you agree with your choices above. |
| Submarine | An onboard explosion has damaged a submarine, killed and injured several crewmembers. Additionally, it has collapsed the only access corridor between the upper and lower parts of the submarine. The upper section, where the most of the crewmembers are located, does not have enough oxygen remaining for all of the crewmembers to survive until they reach the surface. You are located in the lower section by yourself, where there is enough oxygen. There is an emergency access hatch between the upper and lower sections of the submarine. If released, it will fall to the deck and allow oxygen to reach the upper section. However, you were knocked unconscious and are lying beneath the hatch while the rest of the crew are almost out of air. If the crewmembers in the upper section shove the hatch open they will have air, but it will fall to the deck, crushing you to death. Should the crewmembers in the upper deck open the hatch and crush you below to save crewmembers in the upper deck?  Please choose whether you agree or disagree with the suggested action in the scenario.  Please select the extent to which you agree with your choices above. |
| *Self-in-majority* context | |
| Crying baby | Enemy soldiers have taken over a village. They have orders to kill all remaining civilians over the age of two. You and some of the townspeople have sought refuge in two rooms of the cellar of a large house. Outside you and the villagers hear the voices of soldiers who have come to search the house for valuables. A baby, who is with a villager in the room, begins to cry loudly. The parent put his/her hand over the baby’s mouth to block the sound. If the parent removes his/her hand from the baby’s mouth the baby’s crying will summon the attention of the soldiers who will spare the baby’s life, but will kill the parent and the others, including you, hiding in both rooms. To save yourself and the others the parent must keep his/her hand on the baby’s mouth and smother the baby to death. Should the parent smother his/her baby in order to save him/herself, you and the other townspeople?  Please choose whether you agree or disagree with the suggested action in the scenario.  Please select the extent to which you agree with your choices above. |
| Burning building | You and six other people are trapped in a burning building. There is only one emergency exit through which everyone could escape to safety, but it is blocked by burning debris. One of the seven people in the hallway leading to the exit who has been injured is about to crawl to safety through a small hole at the bottom of the exit door. You and the other five people do not have time to climb through the small hole. You and the other five people could grab the injured person and use his/her body as a battering-ram to break through the burning blockage in the hallway that is preventing their escape. Doing this is certain to kill the injured person. However, if you and the other five people do not do this, you and the other five people will die. Should you and the other five people kill the injured person in order to save yourself and the other five people?  Please choose whether you agree or disagree with the suggested action in the scenario.  Please select the extent to which you agree with your choices above. |
| Submarine | An onboard explosion has damaged a submarine, killed and injured several crewmembers. Additionally, it has collapsed the only access corridor between the upper and lower parts of the submarine. The upper section, where you and the most of the crewmembers are located, does not have enough oxygen remaining for all of the crewmembers to survive until they reach the surface. Only one remaining crewmember is located in the lower section, where there is enough oxygen. There is an emergency access hatch between the upper and lower sections of the submarine. If released, it will fall to the deck and allow oxygen to reach the upper section. However, a crewmember in the lower section was knocked unconscious and is lying beneath the hatch while the rest of the crew are almost out of air. If you and the crewmembers in the upper section shove the hatch open they will have air, but it will fall to the deck, crushing the unconscious crewmember to death. Should you and the crewmembers in the upper deck open the hatch and crush the crewmember below to save crewmembers in the upper deck?  Please choose whether you agree or disagree with the suggested action in the scenario.  Please select the extent to which you agree with your choices above. |

**Reference**

Moore, A. B., Clark, B. A., & Kane, M. J. (2008). Who Shalt Not Kill? Individual Differences in Working Memory Capacity, Executive Control, and Moral Judgment. *Psychological Science, 19*(6), 549–557. doi:10.1111/j.1467-9280.2008.02122.x
